# Bolt Installation Defect Detection Based on a Multi-Sensor Method

**DOI:** 10.3390/s23094386

**Published:** 2023-04-29

**Authors:** Shizhao An, Muzheng Xiao, Da Wang, Yan Qin, Bo Fu

**Affiliations:** 1 School of Mechanical Engineering, Beijing Institute of Technology, Beijing 100081, China; 2 Beijing Institute of Remote Sensing Equipment, Beijing 100081, China; 3 Huaihai Industries Group Co., Ltd., Changzhi 046000, China

**Keywords:** bolt installation, YOLO v3, defect detection, multi-sensor

## Abstract

With the development of industrial automation, articulated robots have gradually replaced labor in the field of bolt installation. Although the installation efficiency has been improved, installation defects may still occur. Bolt installation defects can considerably affect the mechanical properties of structures and even lead to safety accidents. Therefore, in order to ensure the success rate of bolt assembly, an efficient and timely detection method of incorrect or missing assembly is needed. At present, the automatic detection of bolt installation defects mainly depends on a single type of sensor, which is prone to mis-inspection. Visual sensors can identify the incorrect or missing installation of bolts, but it cannot detect torque defects. Torque sensors can only be judged according to the torque and angel information, but cannot accurately identify the incorrect or missing installation of bolts. To solve this problem, a detection method of bolt installation defects based on multiple sensors is proposed. The trained YOLO (You Only Look Once) v3 network is used to judge the images collected by the visual sensor, and the recognition rate of visual detection is up to 99.75%, and the average confidence of the output is 0.947. The detection speed is 48 FPS, which meets the real-time requirement. At the same time, torque and angle sensors are used to judge the torque defects and whether bolts have slipped. Combined with the multi-sensor judgment results, this method can effectively identify defects such as missing bolts and sliding teeth. Finally, this paper carried out experiments to identify bolt installation defects such as incorrect, missing torque defects, and bolt slips. At this time, the traditional detection method based on a single type of sensor cannot be effectively identified, and the detection method based on multiple sensors can be accurately identified.

## 1. Introduction

In recent years, the demand for the automatic installation detection of the defects of bolts and other parts has increased significantly. Usually, railway and highway inspection and power transmission line inspection require class detection [1,2,3,4], which involves fastener condition detection and surface defect detection. At the same time, it also has some applications in the field of the automatic assembly of parts. At present, automatic bolt defect detection generally relies on a single type sensor, such as a torque sensor, vision sensor and so on. Most sensors rely on the collected data to make direct judgments, while visual sensors rely on the collected images to form judgments and output results through trained neural networks, such as SSD, YOLO, and so on [5,6,7,8,9,10,11].

For the problem of bolt installation defect detection, manual detection is not applicable in some extreme scenarios, except for the problems of low efficiency, high false detection rates, and inadequate detection rates. In automatic detection, the most commonly used sensors are torque sensors, range sensors, and visual sensors. However, each sensor can only perceive limited information, so the application of a single type of sensor is also prone to false detection or inadequate detection phenomena, as shown in Table 1.

In this paper, in order to avoid the false and inadequate detection of a single type of sensor, we propose a multi-sensor detection method. The torque and angle sensors inside the screwdriver and the visual sensor described in this paper are used for the comprehensive detection and judgment of bolt installation defects. Torque and angle sensors can detect bolt torque defects and whether bolts have slipped, which visual sensors cannot detect. However, vision sensors can detect the incorrect and missing installation of bolts that the torque and angle sensors cannot detect. Finally, with the combination of a variety of sensor judgment results, a comprehensive judgment can be achieved. This method can avoid most of the false and inadequate detection caused by using a single type of sensor.

The remainder of this paper is structured as follows: Section 2 reviews the research status of automatic bolt installation defect detection and the main contributions of this paper. Section 3 describes the detection methods and main processes involved. In Section 4, the realization of visual detection and the results of neural network training are described. Section 5 introduces the experiment of bolt installation defects using multiple sensors, and expounds the importance of using multiple sensors for detection. Section 6 illustrates the main conclusions of the experiment.

## 2. Related Work and Contributions

### 2.1. State of the Art

To improve assembly efficiency, articulated robots have gradually replaced labor in the field of bolt installation. The automatic bolt installation of the manipulator is usually guided by sensors, such as multi-axial force sensors and vision sensors [12,13,14]. Although the installation efficiency is improved, installation defects may still occur, so it is necessary to carry out high reliability bolt installation defect detection. Although many researchers have conducted research on bolt loosening and parameter identification [15,16,17,18], it is still necessary to carry out the automatic identification of bolts. At present, the automatic incorrect or missing installation/defect detection method realized by various sensors has gradually become the mainstream. Zhu et al. [19] used laser-ranging sensors in the production of parts of different vehicle models in the same line and at the same station to avoid the phenomenon of missing or incorrect assembly. Yu et al. [20] invented a device to prevent the absence of bolts in the assembly and manufacturing processes by using torque sensors, which have the characteristics of low requirements for installation layout and a low cost. With the development of visual inspection technology, this has gradually become the mainstream method of bolt absence/defect detection. Liu [21] studied the detection of truck stop keys, and used the gray level co-occurrence matrix of the target image and a support vector machine (SVM) to complete the fault identification process. Feng [22] proposed an automatic fastener fault identification method, and realized the detection of fastener wear and loss by using a probabilistic topic model. Zhong [23] conducted a more in-depth study on the defects of missing pins by constructing an optimized PVANET structure, and the last level of recognition was changed to the detection of multiple local areas. The visual information of multiple areas was integrated to judge the defects of cotter pins, and good results were obtained.

Yang and Marino [24,25] realized the real-time detection of hook fasteners and hexagonal fasteners, respectively. On this basis, Ayteki et al. [26] established a real-time detection system for railway fasteners by using a high-speed laser-ranging camera combined with pixel and histogram similarity analysis, which improved their robustness. Li [27] established a real-time visual inspection system (VIS) for discrete surface defects. This system has the characteristics of a fast running speed and high recognition rate. It can run on a test train of 216 km/h in real time. Resendiz [28] developed a defect detection system for track fasteners based on machine vision, which mainly detects the defects of wooden fasteners and turnout parts through their texture and edges. Kang [29] used a semantic segmentation model to realize the state detection of a railway catenary positioning ring. Souku [30] improved the regularization method of a depth model based on the image data obtained from multi-angle shooting, and obtained good detection results for rail surface defects. Huynh et al. [31] proposed a method to identify the rotation angle of nuts using the Hough transform algorithm and to detect whether the bolt was loose by comparing the angle changes before and after. This method based on visual images can detect the nut rotation angle with an accuracy of ±2.6°.

At present, deep learning is widely used in the field of detection and defect classification [32,33]. Liu et al. [34] proposed a bolt defect identification method that incorporates an attention mechanism and wide residual networks, and the bolt recognition accuracy of this method reached 94.57% compared with the value obtained before the inclusion of the attention mechanism. Zhang et al. [35,36] used Faster R-CNN to train different screw heights after bolt loosening, to determine whether the bolts were tight or loose, and the recognition accuracy reached 95.03%. Sun et al. [37] proposed a bolt loosening detection method based on YOLO v5, which realized bolt-loosening detection by detecting the angle of the bolt relative to the nut.

Pal et al. [38] realized bolt loosening detection by using a convolutional neural network (CNN) to extract recognition features from time–frequency scale images based on vibration. Zhao et al. [39] used SSD to identify the numbers of bolt heads, and calculated the angle between the center coordinates of the two prediction boxes. Pham et al. [40] used the composite bolt images generated by a graphical model as the data set of neural network training, which improved the efficiency of collecting high-quality training data. Qi et al. [41] embedded a dual-attention mechanism in faster regions with a convolutional neural network (Faster R-CNN), to analyze and enhance various visual features at different scales and different locations, which effectively improved the bolt detection accuracy. Li et al. [42] proposed a multi-bolt loosening identification method based on VAM and ResNet-50 CNN, which can identify bolt loosening with reasonable accuracy, computational efficiency, and robustness. CHEN [43] built a three-level defect detection structure based on the SSD and YOLO networks. After positioning the support device, the fasteners were detected. Finally, a separate deep neural network was built for defect identification, and the detection of fastener defects on the catenary support device was completed.

### 2.2. Main Contributions

At present, the automatic detection of bolt installation defects mainly depends on a single type of sensor, which is prone to mis-inspection. In this paper, aiming at the problem of bolt installation defects detection, a detection method based on multiple sensors is proposed. The main contributions of this method are composed of:

1. The torque and angle sensors inside the screwdriver and the visual sensor described are used for the comprehensive detection and judgment of bolt installation defects. Torque and angle sensors can detect bolt torque defects and whether bolts have slipped. Vision sensors can detect the incorrect and missing installation of bolts.
2. Using the YOLO v3 network, the recognition rate of visual detection is up to 99.75%, and the average confidence of the output is 0.947.
3. Simulation experiments are carried out for several single-type sensors which are prone to mis-inspection. The results show that the detection method based on multiple sensors can output accurate detection results in the case of bolt missing or incorrect installation, torque defects and whether bolts have slipped, and has great advantages over the detection method based on a single type of sensor.

## 3. Overview of Detection Methods

**Figure 1:**
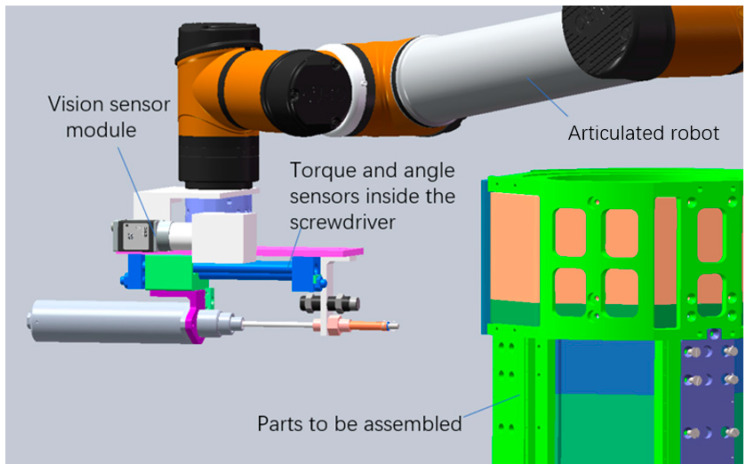
Schematic diagram of the detection device.

The detection device used in this method is mainly composed of a screwdriver module and a vision module, as shown in Figure 1. An angle sensor and torque sensor are built into the automatic screwdriver. The accuracy of the torque sensor is 5/1000. The accuracy of the angle sensor is ±3°. The vision module is composed of a CCD camera, lens and coaxial light source. The maximum resolution supported by the CCD camera is 2592 × 1944, the minimum is 160 × 120, and the focal length is 25 mm. The resolution used for visual inspection is 1280 × 800. The lens adopts an adjustable telecentric zoom lens. The two modules are connected to the end of the articulated robot, and the device can detect the bolt assembly at each position of the parts through the movement of the articulated robot.

**Figure 2:**
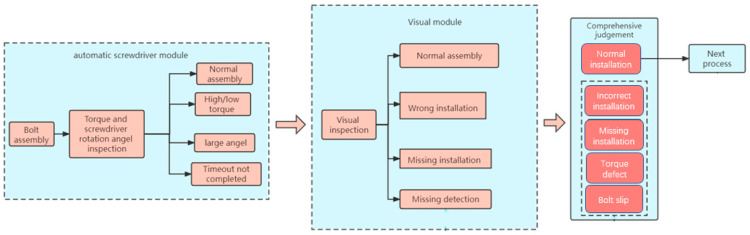
Main process of the detection method.

The main process of the detection method is shown in Figure 2. The sensor in the automatic screwdriver module monitors the torque and angle. The vision module is mainly responsible for identifying the incorrect or missing installation. Combined with the judgment results of the two modules, the bolt assembly status is comprehensively judged. The main judgment logic is as follows:

1. When the inspection results of both modules are normal assembly, the final inspection result is normal assembly of the bolts.
2. When the inspection result of the screwdriver module is a torque defect and the inspection result of the visual module is normal assembly, the final inspection result is that the bolt has a torque defect.
3. When the inspection result of the screwdriver module is normal assembly and the inspection result of the visual module is incorrect installation, the final inspection result is incorrect installation.
4. When the detection result of the screwdriver module is that the torque is too low, the angle is too large and the time is out, and the detection result of the visual module is that the bolt is missing installation, the final detection result is missing installation.
5. When the inspection result of the screwdriver module is too large and the inspection result of the visual module is normal assembly, the final inspection result is the bolt slip.

## 4. Visual Monitoring Based on YOLO v3

### 4.1. Algorithm Overview

**Figure 3:**
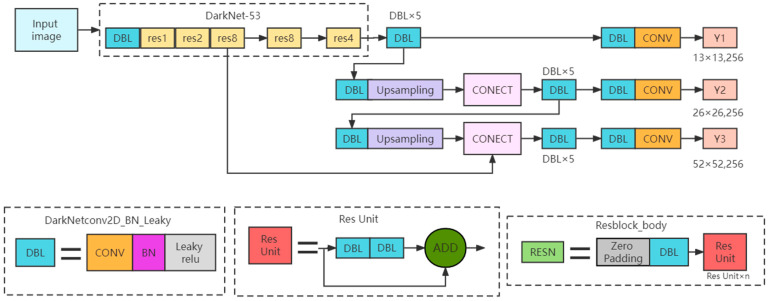
YOLO v3 network structure.

YOLO v3 is a single-stage target detection algorithm, which only uses convolution layers and is classified as a full convolution network (FCN). It mainly improves the network structure, network characteristics and subsequent calculation, and improves the detection accuracy, while ensuring real-time detection [11]. The main structure of the network is shown in Figure 3. The YOLO v3 network absorbs advanced framework ideas, such as feature fusion and residual networks, and proposes DarkNet-53, which contains 53 convolutional layers [11]. A large number of residual connections are used in the basic network to enhance the ability of the model to converge. At the same time, it can output feature maps of 13 × 13, 26 × 26 and 52 × 52 scales, which is conducive to the detection of multi-scale objects and small objects. DarkNet-53 eliminates the pooling layer, of which the down-sampling is achieved by a convolution kernel with a step size of 2.

YOLO v3 follows the anchor mechanism. The feature maps of the three scales correspond to three priori boxes, as shown in Table 2. The K-means algorithm is used for clustering to obtain the nine priori boxes shown in the table, and they are then allocated.

In addition, in order to realize a multi-category prediction, YOLO v3 uses the logistic function instead of the Softmax function. Logistic classification is mainly composed of linear summation, sigmoid function activation, calculation errors and correction parameters. The first two parts are used for judgment, and the last two parts are used for correction. Logistic classifiers can realize the decoupling between categories to ensure the multi-label classification of the target.

After obtaining the anchor box, the coordinates *t*_x_, *t*_y_, *t*_w_, *t*_h_ predicted by YOLO v3 are offsets rather than real coordinates, while the center coordinates bx and by width, and height *b*_w_ and *b*_h_ of the prediction box are obtained by Equation (1). The σ in the formula is the Sigmoid function, which is used to constrain *t*_x_ and *t*_y_ in the range of (0, 1) to prevent an excessive offset of the prediction box, *c*_x_ and *c*_y_ are the coordinates of the upper left corner of the grid, and *p*_w_ and *p*_h_ are the width and height of the prior box.

(1) $$\begin{array}{c} b_{x}=\sigma \left(t_{x}\right)+c_{x}\\ b_{y}=\sigma \left(t_{y}\right)+c_{y}\\ b_{w}=p_{w}e^{t_{w}}\\ b_{h}=p_{h}e^{t_{h}} \end{array}.$$

The loss function of YOLO v3 is shown in Equation (2). Its loss function consists of three parts: center coordinate and the width–height error, confidence error and category error. Compared with the loss function of v2, the difference of v3 is that the confidence loss and category loss are changed to binary cross entropy. For example, in Equation (3), $y_{i}$ is a binary label value 0 or 1, and $p\left(y_{i}\right)$ is the probability of belonging to the $y_{i}$ label value. In Equation (2), the first two behaviors are the center coordinates and width–height errors, the third and fourth behaviors are the confidence errors, and the last behavior category error, *S*^2^, represents the number of grids. *B* represents the number of prediction boxes, *λ*_coord_ is the weight of positioning loss, *λ*_noobj_ is the weight of negative sample loss, and *I_ij_^obj^* represents that on the *i*^th^ grid, the *j*^th^ prediction box has a target, and its value is 1, otherwise it is 0. *I*_ij_^nobj^ means that in the *i*^th^ grid, the *j*^th^ prediction box has no target and its value is 1; otherwise, it is 0. $C_{i}^{j}$ and $P_{i}^{j},$ respectively, represent the confidence predicted value and probability belonging to a certain category of the *i*^th^ grid and the *j*^th^ prediction box.


(2) $$\begin{array}{c} \;\mathrm{Loss}\;=\lambda_{\mathrm{coord}}\sum_{i=0}^{S^{2}}\sum_{j=0}^{B}I_{ij}^{\mathrm{obj}\;}\left[{\left(x_{i}-\hat{x}\right)}^{2}+{\left(y_{i}-{\hat{y}}_{i}^{j}\right)}^{2}\right]\\ \;+\lambda_{\mathrm{coord}}\sum_{i=0}^{S^{2}}\sum_{j=0}^{B}I_{ij}^{obj}\left[{\left(\sqrt{w_{i}^{j}}-\sqrt{{\hat{w}}_{i}^{j}}\right)}^{2}+{\left(\sqrt{h_{i}^{j}}-\sqrt{{\hat{h}}_{i}^{j}}\right)}^{2}\right]\\ \;-\sum_{i=0}^{S^{2}}\sum_{j=0}^{B}I_{ij}^{obj}\left[{\hat{C}}_{i}^{j}\log\left(C_{i}^{j}\right)+\left(1-{\hat{C}}_{i}^{j}\right)\log\left(1-C_{i}^{j}\right)\right]\\ \;-\lambda_{\mathrm{noobj}}\sum_{i=0}^{S^{2}}\sum_{j=0}^{B}I_{ij}^{\mathrm{noobj}\;}\left[{\hat{C}}_{i}^{j}\log\left(C_{i}^{j}\right)+\left(1-{\hat{C}}_{i}^{j}\right)\log\left(1-C_{i}^{j}\right)\right]\\ \;-\sum_{i=0}^{S^{2}}I_{ij}^{obj}\sum_{c\in \mathrm{classes}}\left(\left[{\hat{P}}_{i}^{j}\log\left(P_{i}^{j}\right)+\left(1-{\hat{P}}_{i}^{j}\right)\log\left(1-P_{i}^{j}\right)\right]\right) \end{array}.$$

(3) $$\mathrm{BCELoss}=-\frac{1}{n}\overset{n}{\underset{i=1}{\sum }}\left[y_{i}\cdot \log p\left(y_{i}\right)+\left(1-y_{i}\right)\cdot \log\left(1-p\left(y_{i}\right)\right)\right]$$

**Figure 4:**
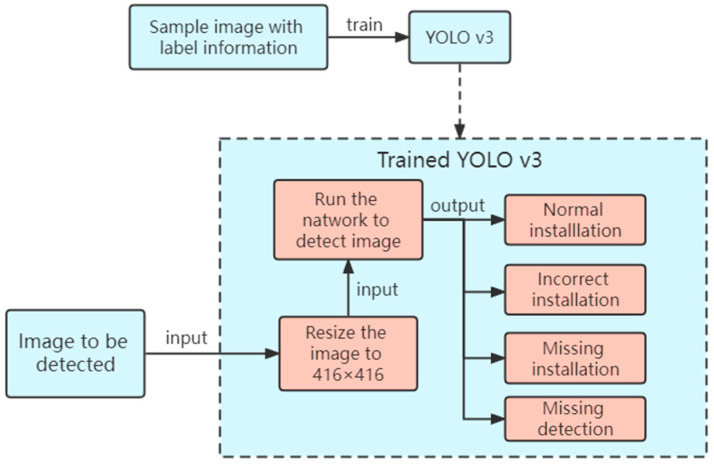
Visual identification of the bolt incorrect and missing installation processes.

Figure 4 shows the complete process for the visual recognition of the incorrect and missing installation of bolts. First, sample images with labels are created and input into the network for training. After obtaining the ideal model, the target image is input into the ideal model, which adjusts the target image to the default size (416 × 416) for detection. Non-maximum suppression (NMS) is used to classify and output the category, confidence, and output box. If no label is detected (that is, no label is output) or the labels are incorrectly assigned to the output bolt, manual detection is performed. If the labels are assigned to the output bolt, the next step is performed.

### 4.2. Network Training

#### 4.2.1. Experimental Data Preparation

**Figure 5:**
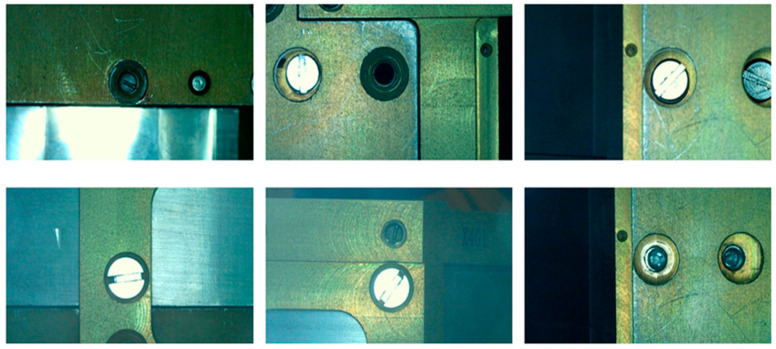
Partial sample example.

A CCD camera and coaxial light source were used to collect images of bolts assembled or not assembled at each part after the parts that were set to be assembled were placed in the frame, and the blurred and repeated invalid samples were removed. In total, 1400 images (resolution 1280 × 800) were obtained, as shown in Figure 5, among which 515 bolt normal assembly images (label: Yes), 267 flat-head bolt missing installation images (label: No), 234 stud bolt missing installation images (label: Nop), 177 flat-head incorrect bolt installation images (label: zcp), and 207 stud incorrect bolt installation images (label: pcz). The obtained images were randomly assigned to the training set, verification set and test set, with the corresponding ratio of 9:1:4.

**Figure 6:**
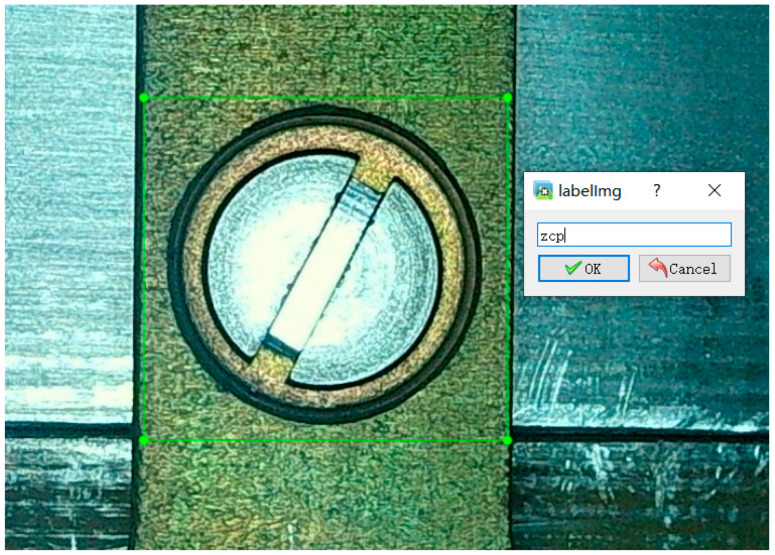
Label interface.

Since the label file input in the YOLO v3 network is XML, labeling in Anaconda was used to annotate the image and generate an XML file with label-type and location information, as shown in Figure 6.

#### 4.2.2. Model Training and Experimental Environments

The computer environment used in the experiment was the Unbuntu 64 bit system, which used the pythoch 1.7.0 deep learning framework to build the YOLO v3 neural network, and the CUDA version used was 11.0, cuDnn 8.0.5 and NVIDIA GeForce RTX3090. The main parameters of the model training are shown in Table 3. The Weight_decay parameter for the training phase was 0.0005, the Batch_ Size was 8, Nms_ Iou was 0.3, confidence was 0.5, and the learning rate was 0.0001.

#### 4.2.3. Analysis Results

**Figure 7:**
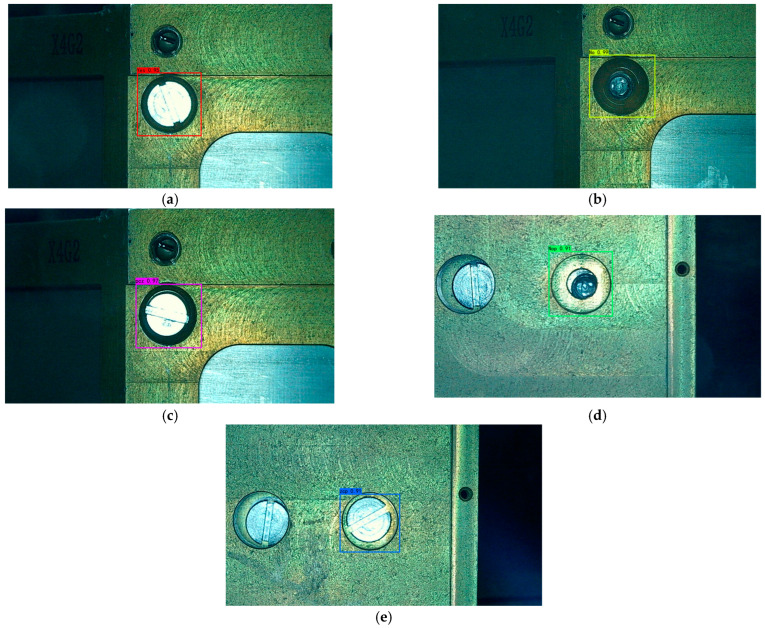
Image recognition effect of some test sets: (**a**) normal assembly image. The recognition result is “Yes”. (**b**) Flat-head bolt missing installation image. The recognition result is “No”. (**c**) Stud incorrect bolt installation image. The recognition result is “pcz”. (**d**) Stud bolt missing installation image. The recognition result is “Nop”. (**e**) Flat-head incorrect bolt installation image. The recognition result is “zcp”.

In the experiment, 100–600 iterations of training were carried out. After the training, the model was verified with the test set image and the recognition effect was observed, as shown in Figure 7.

**Figure 8:**
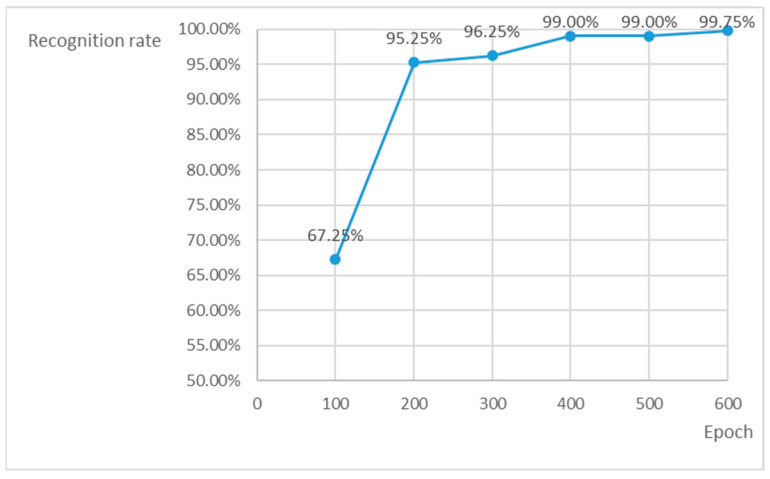
Recognition rate change curve.

**Figure 9:**
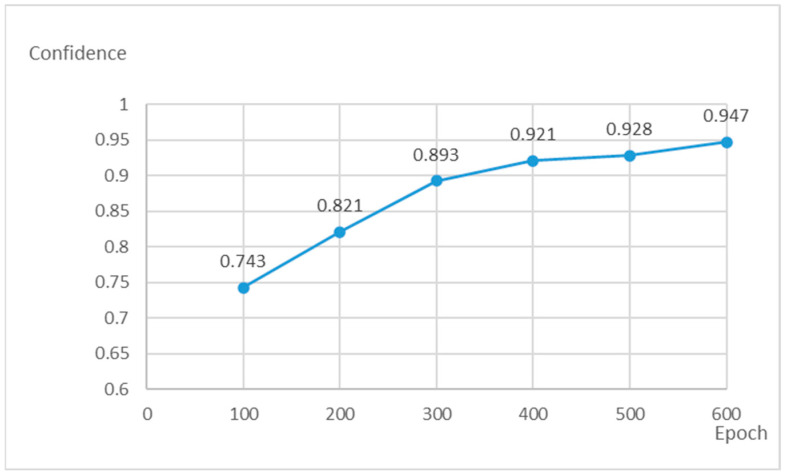
Confidence change curve.

Considering the actual application scenario, the main evaluation indicators of this experiment are the recognition rate (i.e., whether the image information can be output), recognition accuracy (i.e., whether the output image information is correct), and average confidence. The change in the recognition rate with the number of iterations is shown in Figure 8. It can be observed that with the increase in the number of iterations, the recognition rate curve first grows and then tends to be stable, especially after 400 iterations, whereas the recognition rate minimally changes, and the recognition accuracy is more than 99% at this time. The average confidence of the output changes with the number of iterations, as shown in Figure 9. As the number of iterations increases, the confidence curve keeps rising. After comprehensive consideration, the training model with 600 iterations is selected. At this time, the recognition rate is 99.75%, the recognition accuracy is 99.5%, and the average confidence of the output is 0.947. The detection speed is 48 FPS, which meets the real-time requirement.

## 5. Multiple-Type Sensor Detection Experiment

**Figure 10:**
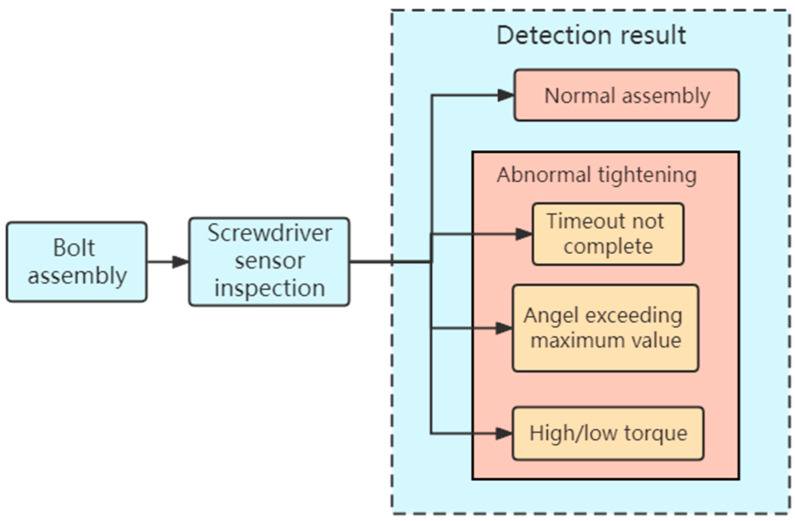
Judgment logic of a torque and angle sensor.

Various types of sensors can effectively detect some assembly defects, as visual sensors can determine the incorrect or missing assembly of different parts, and torque and angle sensors can determine torque and angle defects, as shown in Figure 10. However, through experiments, we know that in some cases, it is difficult to correctly judge the bolt assembly defects by only relying on a single type of sensor. The detection method based on multiple sensors can combine the advantages of various sensors, so that more kinds of assembly defects can be accurately detected.

### 5.1. Detection Subject

**Figure 11:**
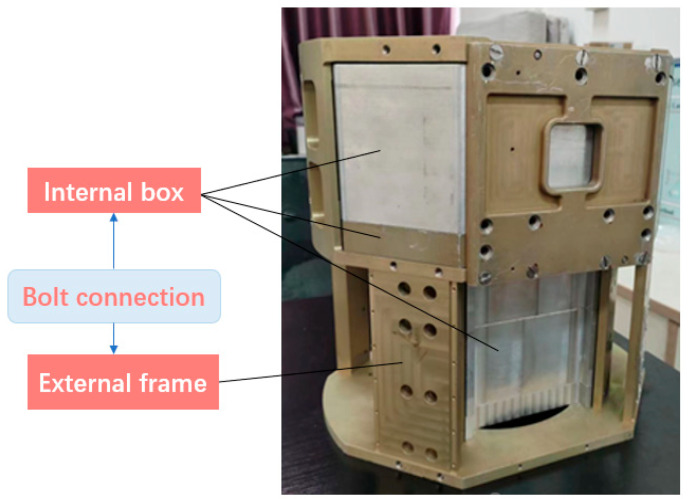
Box-type assembly.

This experiment uses the box assembly shown in Figure 11 for testing, which is composed of an external frame and an internal box, and the two are connected by bolts. This assembly has high reliability requirements for bolt assembly, and assembly defects need to be detected in time, or it will affect the next assembly process.

In the experiment, we use the bolt assembly defect detection method based on multiple types of sensors to detect the incorrect installation, missing installation, torque defects, and the bolts slipped that are prone to false detection by a single type of sensor. The experiment involves the assembly and inspection of flat-head bolts and stud bolts. The rated torque is 4500 ± 100 mN. m, the rated maximum angle is 1200°, and the rated maximum assembly time is 3500 ms.

### 5.2. Experimentation

#### 5.2.1. Normal Bolt Installation

**Figure 12:**
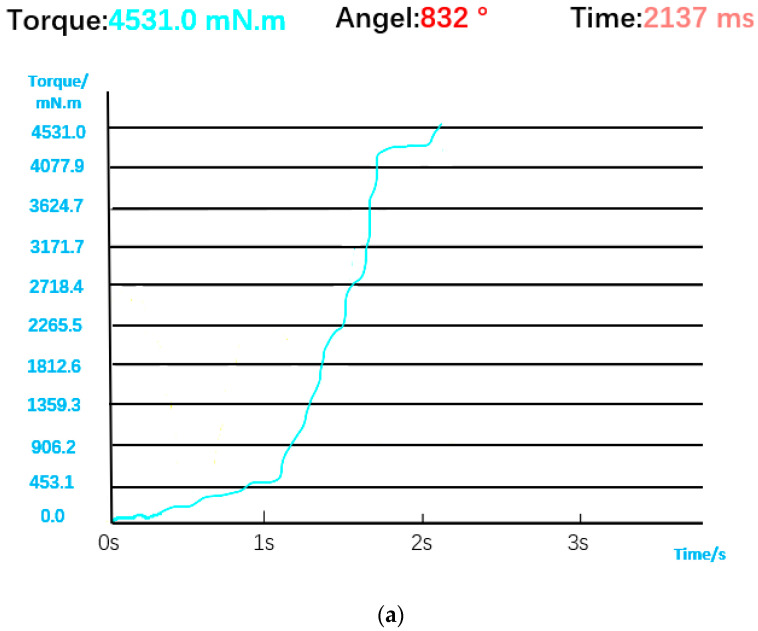

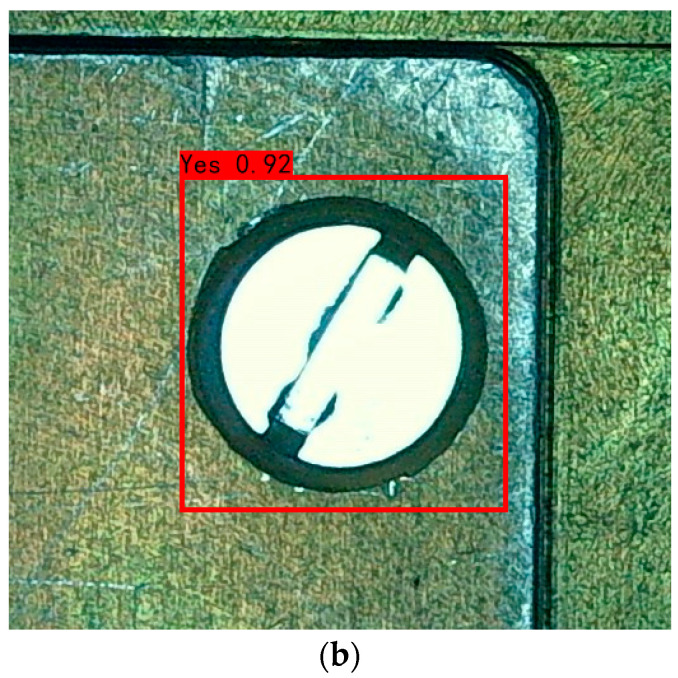
(**a**) Torque and angle data for normal installation. (**b**) Visual judgment result.

**Figure 13:**
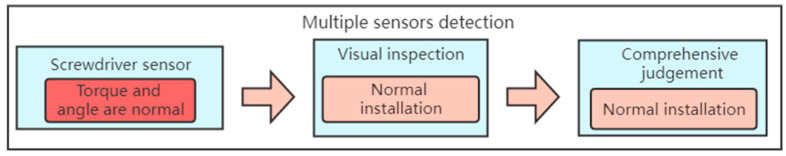
Comprehensive decision logic in the case of normal installation.

The torque and angle data of the bolt during normal installation are shown in Figure 12, and the result of visual inspection is normal installation. The torque curve rises slowly when the bolt is just engaged. After the bolt is buckled, the torque rises rapidly. Since the automatic screwdriver adopts a precise tightening strategy, when the torque is close to the rated torque, the speed becomes slower, and the torque curve will first stabilize and then rise to the rated torque. The comprehensive judgment logic is shown in Figure 13, that is, the bolt installation is considered successful only when the visual detection results and the detection results of the screwdriver sensor are normal installation.

#### 5.2.2. Incorrect Bolt Installation

**Figure 14:**
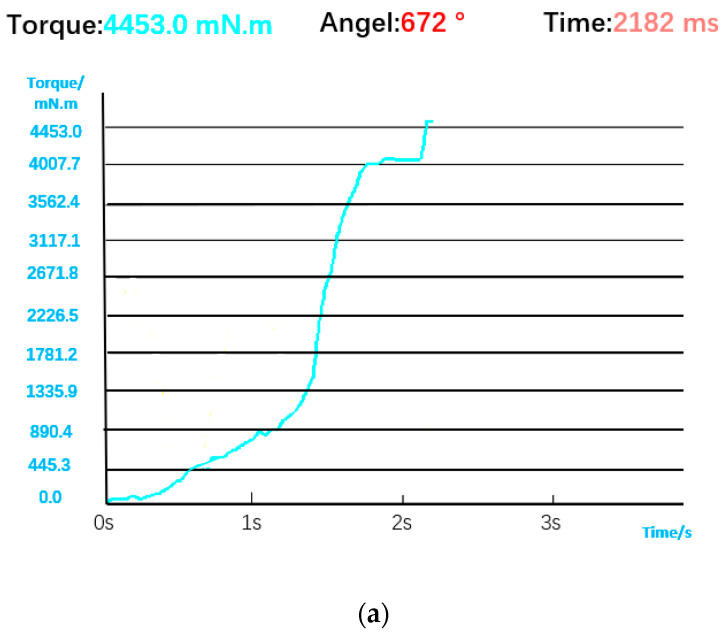

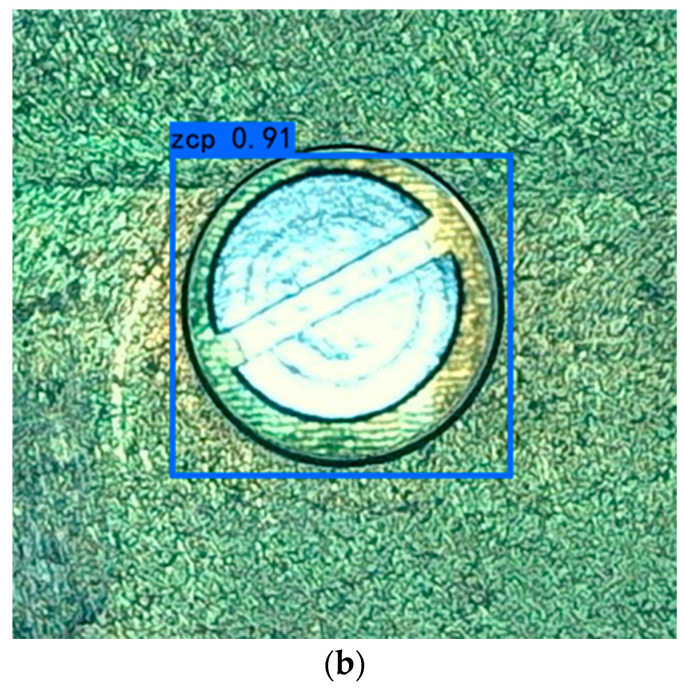
(**a**) Torque and angle data for incorrect installation. (**b**) Visual judgment result.

**Figure 15:**
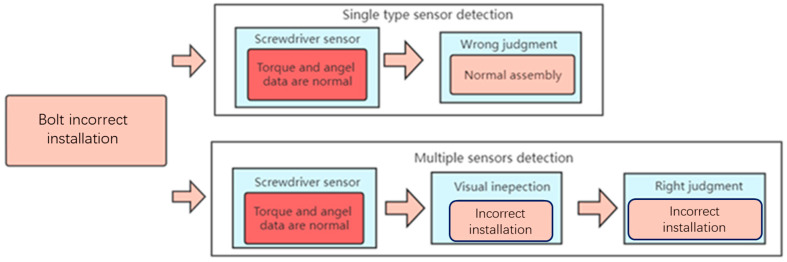
Comprehensive decision logic in case of incorrect installation.

When two bolts with similar calibration torques are incorrectly installed, the torque and angle sensors alone cannot be effectively detected, and the incorrect judgment results of the normal installation will be generated. The torque curve is similar to that of normal installation. The visual sensor will correctly identify the incorrect bolt installation through the YOLO V3 network, as shown in Figure 14. Comprehensive judgment logic is also shown in Figure 15. 

#### 5.2.3. Missing Bolt Installation

**Figure 16:**
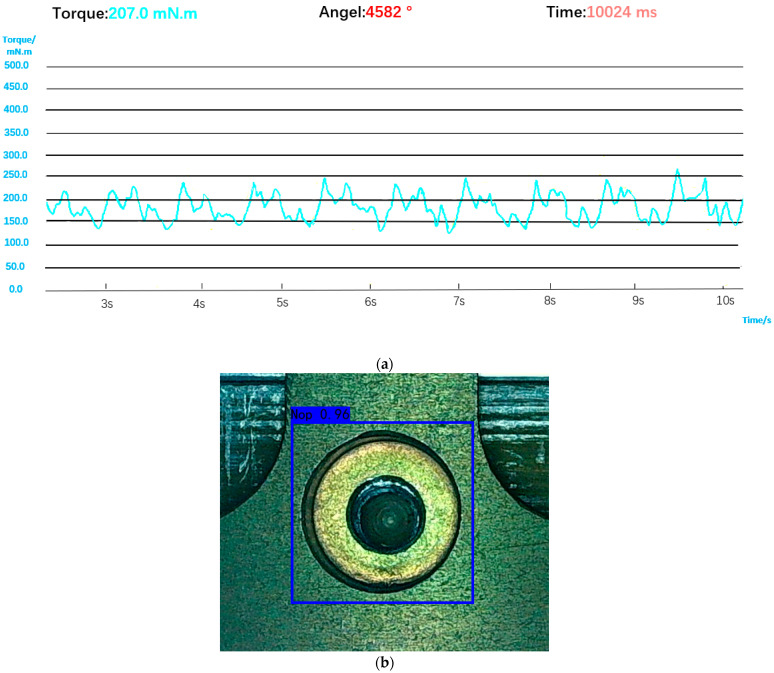
(**a**) Torque and angle data of the screwdriver head idling in the bolt hole. (**b**) Visual judgment result.

**Figure 17:**
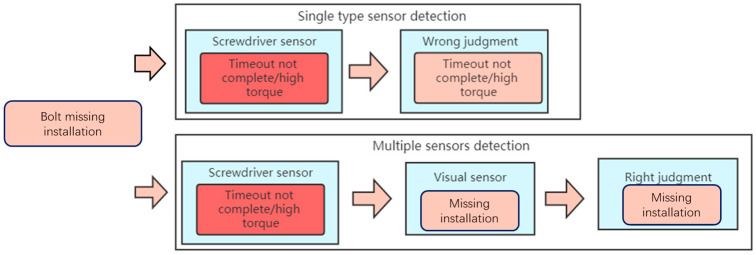
Comprehensive decision logic in case of missing installation.

A screwdriver head will idle in the hole when the screwdriver does not reach the bolt or the bolt falls during the movement. The inadequate installation of the bolts cannot be determined by the torque and angle data alone. At this time, the torque will always be low and the angle data will be too large, as shown in Figure 16. Since there is no bolt screwed in, the torque curve oscillates back and forth in a low torque interval. The data at this time are similar to the data obtained when the bolt does not fall, but the tightening time increases, and it will be wrongly judged as exceeding the tightening time rather than detecting the inadequate installation of the bolt. The visual sensor can correctly identify the missing state of the bolt, as shown in Figure 16. Comprehensive judgment logic is also shown in Figure 17.

#### 5.2.4. Torque Defects and Bolt Slips

**Figure 18:**
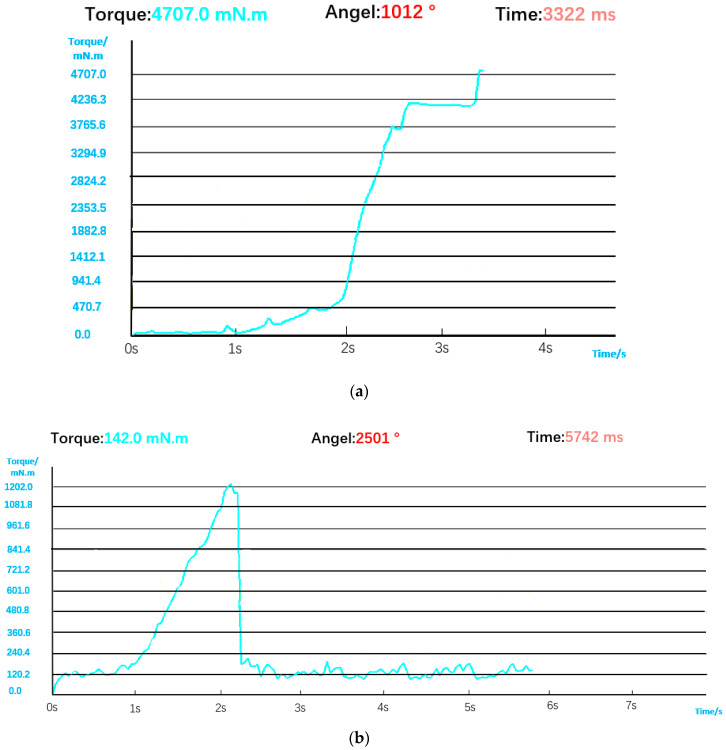

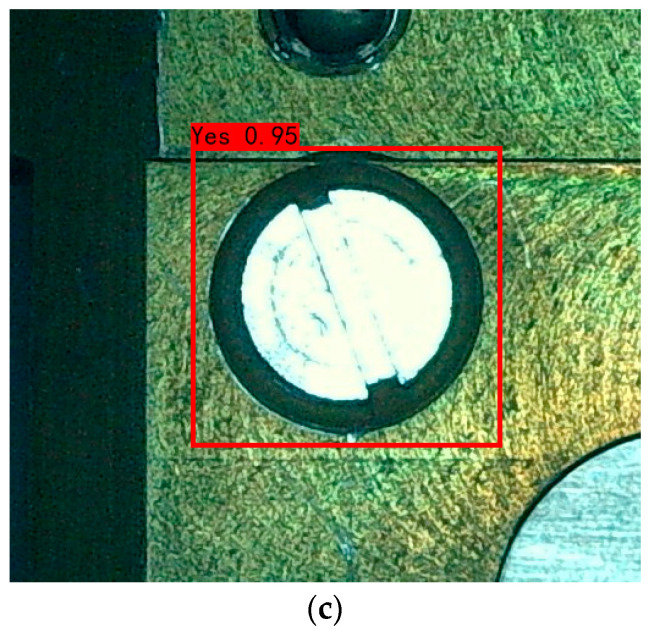
(**a**) Torque and angle data when the torque is too high. (**b**) Torque and angle data when bolt is stripped. (**c**) Visual judgment result.

**Figure 19:**
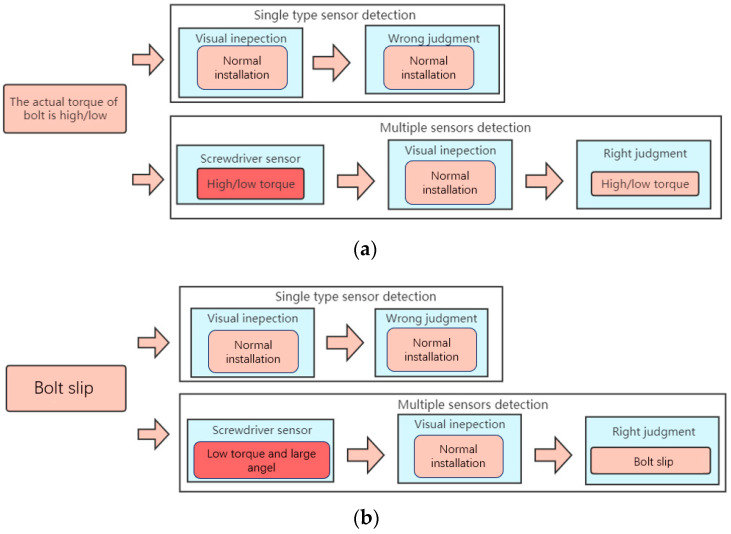
(**a**) Comprehensive decision logic in case of high/low torque. (**b**) Comprehensive decision logic in case of bolt slips.

However, when only visual inspection is used, because it cannot identify torque and angle information, it appears as if the bolt has been assembled, resulting in a false judgment when the bolt has slipped (i.e., the angle is too large) or the torque is too high/low. The torque curve in Figure 18a is similar to that in normal installation on the whole, but in the end, it exceeds the rated torque. The first half of the torque curve in Figure 18b is similar to that of normal installation, but the second half of the torque curve drops rapidly and oscillates back and forth in the low torque interval due to a bolt slip. At this time, the torque and angle sensors can directly detect the two situations, so the visual detection results still need to be corrected by combining torque and angle data. Comprehensive judgment logic is shown in Figure 19.

## 6. Conclusions

In order to solve the problem of the low efficiency and high false detection rate of bolt installation, this paper proposes a method of bolt installation defect detection based on the composite monitoring of multiple types of sensors, which avoids the problem of misjudgment when only a single type of sensor for detection is used. In this paper, the torque and angle sensors inside the screwdriver and the CCD camera are used for a comprehensive detection and judgment.

Vision sensors can detect the incorrect and missing installation of the bolts that the torque and angle sensors cannot detect. Based on YOLO v3, the recognition rate of visual detection is as high as 99.75%, and the average confidence of the output is 0.947. At the same time, torque and angle sensors can detect bolt torque defects and whether bolts have slipped, which visual sensors cannot detect. Through the comprehensive judgment of the detection results of multiple sensors, more accurate judgment results can be obtained, avoiding the misjudgment of a single-type sensor. In short, this method can further improve the reliability and efficiency of detection, meet the real-time requirements, and avoid a series of problems during manual detection.

## Figures and Tables

**Table 1:** Comparison of various types of sensors.

Contrast Term	Torque Sensor	Range Sensor	Visual Sensor
Detection content	Incorrect and missing installation, torque defect	Missing installation	Incorrect and missing installation
Conditions of use	No harsh condition	No harsh condition	Needs steady light
Robustness	Stronger	Stronger	Susceptible to light conditions

**Table 2:** Allocation of prior frames on feature maps.

Characteristic Map Size	Receptive Field	Anchor Box
13 × 13	Large	(116 × 90) (156 × 198) (373 × 326)
26 × 26	Medium	(30 × 61) (62 × 45) (59 × 119)
52 × 52	Small	(10 × 13) (16 × 30) (33 × 23)

**Table 3:** Main training parameters of the model.

Parameter	Numerical Value
Weight_decay	0.0005
Batch_ Size	8
Nms_ Iou	0.3
Confidence	0.5
Learning rate	0.0001

## Data Availability

Not applicable.
